# Growth, Endocrine Features, and Growth Hormone Treatment in Noonan Syndrome

**DOI:** 10.3390/jcm11072034

**Published:** 2022-04-05

**Authors:** Jovanna Dahlgren, Cees Noordam

**Affiliations:** 1 Department of Pediatrics, University of Gothenburg, 41685 Gothenburg, Sweden; jovanna.dahlgren@gu.se; 2 Centre for Paediatric Endocrinology Zurich (PEZZ), 8006 Zurich, Switzerland; 3 Department of Pediatrics, Radboud University Medical Centre, 6525 GA Nijmegen, The Netherlands

**Keywords:** Noonan syndrome, growth, endocrinology, growth hormone treatment, children

## Abstract

Noonan syndrome is a heterogeneous congenital disorder. The main features are typical facial features, short stature and cardiac defects. The diagnosis is clinical: in 80% of patients with Noonan syndrome a genetic defect can be shown. Inheritance is predominantly autosomal dominant and seldom autosomal recessive. In 2001, PTPN11 was the first gene connected to Noonan syndrome, and until now, at least 20 other genes have been discovered. All genes code for proteins involved in the RAS-MAP-kinase pathway, and therefore, Noonan syndrome is one of the known RASopathies. Other RASopathies include neurofibromatosis and CFC syndrome. Short stature is one of the defining features of Noonan syndrome. The cause is not fully understood but is multifactorial. Other endocrinological features are confined to delayed puberty and hypogonadism in boys and males. To increase adult height, children with Noonan syndrome have been treated with human growth hormone since the 1990s. This seems to be beneficial in most of the children treated. In this narrative review, we describe the current knowledge on growth, endocrinological features and growth hormone treatment in patients with Noonan syndrome.

## 1. Introduction

Noonan syndrome (NS) is a heterogeneous congenital disorder with a prevalence of about 1:1000 to 1:2500 [1]. The main features are typical facial features, short stature and cardiac defects [2,3]. The diagnosis is clinical, although in 80% of patients a genetic defect can be detected. Inheritance is predominantly autosomal dominant and seldom autosomal recessive. The condition is familial in less than 50% of cases, and in the other cases, a spontaneous mutation has occurred. In 2001, PTPN11 was the first gene connected to NS, and until now, 20 other genes have been discovered [4]. Two genes are most frequently found (PTPN11 around 50% and SOS1 around 10%) [4]. All genes code for proteins involved in the RAS-MAP-kinase pathway, and therefore, NS is one of the known RASopathies. Other RASopathies include neurofibromatosis, Noonan syndrome with multiple lentigines, CFC syndrome and Costello syndrome. Short stature is one of the main features of the syndrome and is most evident from pre-school years since size at birth is normal, and short stature develops after the infancy period. The endocrine cause is not fully understood but is multifactorial with disturbances at the level of the hypothalamic–pituitary growth hormone axis, a diminished growth hormone sensitivity and disturbances at the level of the growth plate. Other endocrine features are foremost confined to delayed puberty and hypogonadism in boys and males. To increase adult height, children with NS have been treated with growth hormone since the 1990s. This seems to be beneficial in most of the children treated. In this narrative review, we describe the current knowledge on growth, endocrine features and growth hormone treatment in NS.

## 2. Growth

Birth weight and length are reported as normal in population-based cohorts [5,6] but can be subnormal in cohorts eligible for growth hormone therapy, foremost in children with mutations in PTPN1 and RAF1 [7,8]. During the first year of life, growth rapidly declines. At the end of the first year, the mean length is on the third centile. It has been hypothesized that growth during the first year of life is influenced by feeding problems or cardiac disease. While both are frequent in NS, Croonen et al. [9] showed that growth during the first year was only partially influenced by feeding and cardiac problems; genetic aspects could have a greater impact during this period. Mouse models expressing PTPN11 mutations show a decreased body length upon weaning [10]. After the first year, the mean height of children with NS is around the third centile. Children with NS enter puberty later and reach their adult height later than normal children as their bone age is retarded by approximately 2 years. A genotype–phenotype relationship can be observed as children with mutations in PTPN-11, KRAS and RAF1 are reportedly shorter than children with mutations in SOS1 or BRAF [7,8]. Adult height has been reported at around 162.5 cm in men and 153 cm in girls [6,8,11]. In adult cohorts of persons with NS measured after age 24, foremost in males, adult height was significantly higher [12,13]. In an unpublished Dutch cohort (71 persons) mean adult height, measured after age 24, was around 169 cm in men and 156.5 cm in women, confirming the findings of Binder et al. (unpublished data, Noordam). This illustrates that people with NS, especially males, tend to grow until their early twenties. This should be considered when judging the effect of growth hormone therapy on final height [14].

## 3. Endocrine Features

### 3.1. Growth Hormone–IGF-1 Axis

As people with NS show proportionate short stature, the growth hormone–IGF-1 axis has been studied. The number of studies, as well as the study samples, is rather small. In these studies, growth hormone deficiency has not been shown convincingly [15,16]; however, neurosecretory deficiency has been shown in small cohorts [17,18]. The IGF-1 levels of people with NS have been shown to be low and could be influenced by retarded puberty or by growth hormone resistance [19,20]. Over the last decade, with increasing knowledge of molecular biological mechanisms, we have gathered more insight into the possible mechanisms underlying disturbances in growth hormone secretion, growth hormone resistance and growth plate disturbances. It has been shown that hypothalamic–pituitary genes of the RAS-MAP kinase pathway are involved in the development of the hypothalamic–pituitary axis [21], which suggests that the hypothalamic–pituitary architecture could be deranged in RASopathies. The previously reported neurosecretory deficiency could be explained by these changes in hypothalamic–pituitary morphology. Binder suggested that decreased growth hormone sensitivity, caused by activation of SHP-2 and subsequently decreased JAK-STAT activity, results in post-receptor growth hormone insensitivity [19]. Accordingly, in IGF-1 generation tests, a decreased response of IGF-1 to growth hormone has been shown [22]. Inhibition of the RAS-MAP kinase pathway by newly developed drugs was able to restore IGF-1 production and partially restore normal growth, in mice [23]. In growth plate hormones, paracrine factors and genes interact for normal growth. Altered growth and architecture of the growth plate have been shown to occur in RASopathies from fundamental studies [24,25].

### 3.2. Gonadal Axis

Puberty typically is delayed by about 2 years for people with NS. Age at menarche is reported as almost 15 years [12,26]. Mean age at onset of puberty in boys was reported at 13.5 years [27], at 13.4 years [15], at 13.2 years [28] and at 14.5 years [13]. Few papers reported that the onset of puberty was more often delayed in girls than in boys: 49% in girls vs. 28% in boys [29] of which 44% of the girls entered puberty after the age of 13 years [30]. The latter study suggests an effect of low body weight on the delay of puberty [29]. There are no reports on fertility disturbances in females with NS, suggesting fertility is normal. Testosterone levels were normal in males, but there is increasing evidence for Sertoli cell dysfunction. Sertoli cell dysfunction was reported in a small cohort of boys with Noonan syndrome and normally descended testes, with decreased AMH and Inhibin-B levels [31]. These findings were confirmed in larger cohorts [27,32]. The reported Sertoli cell dysfunction was independent of cryptorchidism, which is frequent in NS, or orchidopexy. Despite the delayed puberty, only a minority of the children with NS need a pubertal induction [13]. Given the Sertoli cell dysfunction, one could expect reduced fertility in men with NS. Data on fatherhood in people with NS are limited. Ranke reported a preponderance of maternal transmission in non-sporadic cases [6].

### 3.3. Thyroid

Thyroidal antibodies have been reported in NS but overt hypothyroidism is apparently not more common than in the normal population [26,33,34]. Autoimmune diseases, such as autoimmune thyroiditis as well as systemic lupus erythematosus, celiac disease, autoimmune hepatitis and vitiligo, have been reported [35].

### 3.4. Adrenal

No data exist to suggest that there is any problem with adrenal function. Adrenarche, as well as gonadarche, seems to be delayed for people with NS [36].

## 4. Growth Hormone Treatment

### 4.1. Effect of Treatment

Studies into the effects of growth hormone therapy in NS started after the success of growth hormone therapy in people with Turner syndrome in the early 1990s. In the beginning, there were some anecdotal reports with unequivocal results [37,38]. From these early years, two partially controlled studies took place. A study by MacFarlane et al. [39] showed a gain of 3.3 cm in comparison with untreated children after 3 years of growth hormone therapy [39]. Noordam et al. showed an increment of +0.5 in height SDS over one year of growth hormone therapy compared to controls [40]. There are, as in virtually all conditions treated with growth hormone, no long-term controlled studies evaluating the effect of growth hormone therapy on final height in NS. Therefore, we must base our evidence of the efficacy of growth hormone therapy in NS on the available observational studies.

Over the years, more studies on the effect of growth hormone therapy on the final height of children with NS have been conducted with larger sample sizes [28,41,42]. A few of these studies were post-marketing studies [30,42,43]. The post-marketing studies are of interest for providing more data on the safety of growth hormone therapy due to the large number of participants involved. Certainly, all studies differ in design, dosing, and patient characteristics, but the common denominator is an increment in final height for national standards of approximately 1.4 SD, with a broad range (SD 0.8) [44]. Duration of growth hormone therapy and years of therapy before puberty seem to have a positive effect on final height [28,30,41]. Based on this evolving evidence and two specific studies, short stature in NS is an approved indication for growth hormone therapy [28,45].

Findings on growth hormone doses are inconclusive. In most studies, growth hormone dose is, based on prior expertise from Turner syndrome, approximately 45–50 microgram/kg/day. Osio et al. compared 2 doses (33 and 66 microgram/kg/day) and did not find a significant difference [28]. The treatment duration, especially in the prepubertal years, was clearly superior to other studies. As there is a genotype–phenotype relation regarding height in NS, one could expect a better response to growth hormone therapy in children with mutations in genes other than PTPN11 and KRAS. This could not be shown convincingly due to the smaller numbers and the preponderance of children with PTPN11 in the studies. It should be noted that final height was clearly below the target height, but the majority of children reached a height in the normal range (>−2 SD).

### 4.2. Adverse Events and Safety

From the available studies and reports on growth hormone therapy for NS, the findings on adverse effects and complications are reassuring. Non-serious adverse events, which may be encountered during growth hormone therapy, such as headaches, pain or erythema at the injection site, are not frequently reported [30,42]. Scoliosis is reported as it is presumably more prevalent in NS as compared to other conditions. Two areas of interest include cardiovascular complications and neoplasms given that cardiac malformations are prevalent in NS and people with NS have an increased risk for developing cancer.

There have been no reports of hypertrophic cardiomyopathy developing or increasing during growth hormone therapy. Of note, pre-existent hypertrophic cardiomyopathy was an exclusion criterium in most studies. There are a few, personal (unreported) communications: one study included some participants with NS and hypertrophic cardiomyopathy that did not show signs of hypertrophic cardiomyopathy progression during growth hormone therapy [46]. Two studies specifically studied ventricular wall thickness during GH therapy without any signs of increased growth of the ventricular wall [16,47]. Regardless, it is recommended to monitor ventricular wall thickness during growth hormone therapy. Growth hormone therapy is not recommended in children with mutations in genes connected to an increased risk for developing hypertrophic cardiomyopathy (RIT1 and RAF), particularly before the age of 4. This is because in most cases the hypertrophic cardiomyopathy develops before the age of 4 years [48]. In the large post-marketing studies previously discussed, cardiovascular complications have been reported: cardiac hypertrophy, cardiac arrhythmias and pulmonary stenosis [30,49]. It was not possible to prove a connection to growth hormone therapy in these studies.

People with NS have a slightly increased risk for developing cancer compared to the general population (risk is 3.5–8 times increased) [50,51]. Specific mutations in PTPN11—e.g., p.Asp61 and p.Thr73lle—are associated with an increased risk of developing juvenile chronic myelomonocytic leukemia, which is self-limiting in most cases but can develop into acute myeloid leukemia [52]. Other more frequently observed malignancies in Noonan syndrome are brain tumors and neuroblastoma. Increased growth or development of brain tumors have been reported in some studies [42,53,54]. Still, the incidence of these events could be compatible with the increased risk for developing cancer in NS. To summarize, there is no evidence for an increased risk of developing cancer during growth hormone therapy in NS, but there is also no proof that this risk in absent. This question is not confined to NS but is also an issue in other conditions treated with growth hormone. There remains a lack of evidence from studies in the given conditions that cancer risk is increased during growth hormone therapy [55]. Growth hormone therapy in children with specific mutations known to increase risk for developing leukemia is not recommended before the age of 5 years, as this is the period when they are at risk for developing leukemia [56].

## 5. Discussion

Endocrine features in NS are confined to disturbances in the growth hormone–IGF-1 axis and to delayed puberty, leading to short stature in most children with NS during the growth phase and in adult life. The etiology of short stature is multifactorial, with the short-term effect of delayed bone maturation and the long-term effects of disturbances at the level of the hypothalamus (secretion), in the growth hormone signaling pathway (resistance) and at the growth plate (decreased growth potential). Available evidence favors a significant effect of GH therapy on adult height for patients with NS without significant side effects. Therefore, observations of growth in children with NS are necessary, and evaluations for secondary growth disorders are recommended in case of a declining growth curve or growth below the third centile (national standards). If other causes have been excluded and IGF-1 levels are very low, (below 2SDS) growth hormone testing is still recommended. Given that a clear relation of growth response to growth hormone secretory capacity is lacking, one could argue that growth hormone testing is not necessary. Still, very low IGF-1 levels warrant this evaluation in order to exclude pituitary disease.

We believe growth hormone therapy should be discussed with all children with NS and their families. The appropriate age for starting growth hormone therapy is the age of four years in most children, although extremely short stature can be a reason to start earlier. On the other hand, it has been suggested to start growth hormone therapy after the age of five years, as hypertrophic cardiomyopathy mostly develops before this age. However, despite being very rare, children older than five years of age are not completely protected from this condition, as a few cases of hypertrophic cardiomyopathy developing after growth hormone therapy have been observed in children with NS at 6–8 years of age (Dahlgren, personal communication). Despite this, it is not recommended to postpone growth hormone therapy as prepubertal years are very important for the effect on final height. Based on the studies reported, a typical growth hormone dose would be around 50 microgram/kg/day. A minority of young prepubertal children with low IGF-1 and abnormal growth hormone test results should instead be started with 33 microgram/kg/day. Exclusion criteria are specific mutations in PTPN1 connected to leukemia and mutations in genes connected to the development of hypertrophic cardiomyopathy (e.g., RIT1).

The fact that these specific mutations have consequences for potential complications implies that genotyping is necessary prior to GH therapy. Follow-up during GH therapy should consist of the normal laboratory tests performed during growth hormone therapy in other conditions. Additionally, monitoring with cardiac ultrasound for potential effects on the heart should be performed with minimal frequency during the first years of growth hormone therapy. Given the low a priori chance of developing cancer, surveillance is not recommended. In some boys, it can be necessary to induce puberty for psychosocial reasons when the burden becomes too great. Typically, one would induce puberty with a short course of testosterone (six intramuscular injections of 50–100 mg monthly).

As the total number of children with NS treated with growth hormone is still relatively low and there are questions to be solved, careful monitoring of the therapy and, ideally, pooling the data in a nationwide registry is recommended. Only in this way will be able to confirm that growth hormone therapy is safe for the heart, cancer development and metabolic effects. In addition, genotype response relations can be investigated, enabling specific counselling of children and their parents. Further elucidation on the mechanisms determining the diminished growth of children with NS as well as exploring possible novel therapies with small molecules e.g., modulating the RAS-map kinase pathway, is imperative.

## 6. Conclusions

Short stature is frequent in NS. The etiology is multifactorial and only partly explained by disturbed growth hormone secretion and growth hormone resistance. Growth hormone therapy improved final height within the normal range in most children with NS. There are no clear indications that growth hormone therapy is not safe for NS. Further data on the effect of GH therapy related to genotype and on the safety of treatment are needed.

## Data Availability

Not applicable.
